# Remarks on Delirium Tremens, *Comprising a Rejoinder to the* “*Answer of* Dr. B. Horner Coates *to* Dr. Klapp”

**Published:** 1839-02-09

**Authors:** W. H. Klapp

**Affiliations:** Philadelphia


### REMARKS ON DELIRIUM TREMENS,

*Comprising a Rejoinder to the "Answer of* Dr. B. HORNER COATES *to* Dr. KLAPP."

To the Editors of the Medical Examiner.

*Gentlemen*,—The communication with which Dr. Coates has thought fit to favour you, contained in the last number of the Examiner, in reply to some remarks of mine, which you had the kindness to publish on the 26th of January, I confess has surprised me much, and has no doubt produced a similar impression on the minds of the generality of your readers. In perfect sincerity, I can assure you, that I have ever cherished a goodly degree of respect for that gentleman, both as a physician and a writer; and I did expect that the liberty I had taken with him, would have been received with the liberal feelings of a man of science. But in this respect I have been greatly disappointed; his manner and style, throughout, manifesting, that great offence had been given—although, it will be conceded by all, that my communication was couched in language both courteous and respectful. Dr. Coates' remarks, contained in his lecture, with reference to my practice in the Moyamensing Prison, were very exceptionable, because I understood them to convey an impression that a large portion of the cases I had successfully treated by emetics, actually were not cases of delirium tremens, but of recent drunkenness. That this was the Doctor's meaning, and that he wished so to impress the minds of others, and thereby, as I supposed, to underrate the value of the method of cure used by me, I think, is fully evinced by the language used by him. The *three* following sentences (not *two*, as he states) comprehend *every word* he has said about me and my practice. "I am informed that Dr. Wm. H. Klapp employs emetics, very successfully, in the Moyamensing Prison. I understand his cases include a large portion of instances of recent drunkenness. For this I have used emetics with much advantage, *but not for delirium with phantoms*." The conclusion of the last sentence shows clearly, that the Doctor's intention was to convey an impression that the disease in which emetics were found useful by me, was quite different from genuine delirium tremens, or delirium with phantoms—consequently, that I had committed the gross error, of attributing an efficacy to emetics in delirium tremens, which was, in reality, due to an entirely different affection. It is of such a misrepresentation as this that I have complained. It is this which induced me to say, in my first communication, that Dr. Coates' remarks implied the commission by me of "an error in diagnosis." I, of course, felt desirous that his erroneous statement should be pointed out, and receive that public correction which it deserved.

The annexing, by Dr. Coates, of the word "severe" with "the rule" of our prison, is not correct, because it imports an undeserved implication. If *I* had coupled with the rule or discipline any phrase, to indicate the estimation in which it was held by me, it would have been such a word as *excellent*. But the Doctor, no doubt, will be ready to say, the restriction from the use of spirituous drinks engenders in the prison cases of delirium tremens, and this is his reason for calling the rule "severe." But is the Doctor ready to recommend the alternative, of extending to the new comers to the prison the use of spirituous drinks? He is, I suspect, too staunch a moralist for this.

If I apprehend the Doctor rightly, he has thrown out a hint of my being chargeable with a want of frankness in my intercourse with him. He says, "when I parted from Dr. William H. Klapp, in a conversation a few days since, upon the subject of his practice in delirium tremens, I was far from imagining that any appeal to the public was in contemplation, or, indeed, that any offence had been given. Indeed, I understood that gentleman to invite me to visit his patients at the Moyamensing Prison with him." Now, the fact is, that at the time of the conversation alluded to, I was entirely ignorant of the Doctor's having delivered a lecture at the Pennsylvania Hospital on delirium tremens,—the number of the Examiner containing the report of it not having been delivered at my office until the evening of the day on which the conversation was held. If, at that time, I knew nothing, either of the Doctor's lecture or of its errors, may I not also use the Doctor's invocation, and say, "in the name of common sense," how could I, at the time of the conversation, have in contemplation an appeal to the public? Thus, considering myself acquitted from the Doctor's implied charge of a want of ingenuousness in personal intercourse, may I be permitted to ask, how stands the case with respect to the Doctor himself, in this respect? Whose conduct most betrayed a want of candour or frankness—mine, who, at the time referred to, could not possibly have harboured or disguised an intention of calling on him publicly to retract his errors, or his, who, at the very time that I was assuring him that the cases treated at the prison were not cases of intoxication, but genuine delirium tremens, was sending forth to the public an entirely different statement?

To the account which I have given of the nature of the delirium at the prison, the Doctor has added something, which is not correct. He says that they must be considered as "first attacks, and must differ widely from the seventh, fourteenth, and twentieth attacks, so frequent at our hospital." But the fact is otherwise, many of our patients, imprisoned for various grades of crime, are well known, after being discharged, to be brought back every now and then, and nearly as often to become, after some days' confinement and restriction, the subjects of fresh and repeated attacks of the disease. This is especially the case with vagrants, who are committed by our magistrates for thirty days, and who are, many of them, seldom long absent from the prison before they are again returned.

Again, Dr. Coates impliedly charges me with the indelicacy of comparing the success of my own practice with that of my neighbours. Had I done this vauntingly, or with the view to the publicity of my own reputation for success, the charge might be considered as having weight. But the Doctor himself was the first that published the success of my practice, by mentioning it in his lecture, and as he had done so, I conceived there could be no impropriety or indelicacy in stating, in what he chooses to call my appeal to the public, its precise extent. According to my apprehension, the best and only correct way of determining the comparative value and superiority of the different methods of practice, not merely on this subject, but upon all others, is to make a fair estimate of their respective successes, and thus arrive at the knowledge of the excellence of one method over another; this I call medical science, not medical vanity, as the Doctor wishes to represent it.

Feeling conscious of having trespassed much on your polite indulgence, and as the only reparation I can at present make for having occupied so much of the time and attention of your readers, I now propose, most respectfully, to submit to them the history of two cases of delirium tremens, trusting that the change from a subject, in a great measure of a personal character, to that of which I conceive the most successful method of treating a formidable, and often fatal, disease, will prove to them an acceptable offering. In doing this, however, I am aware that I shall expose myself to the reflection of Dr. B. Horner Coates, and, probably, will "be placed (by him) in the somewhat undignified posture of a man comparing the success of his own practice with that of his neighbours."

These cases, it is expected, will not only prove illustrative of the emetic treatment, but at the same time exhibit the characteristic symptoms of the kind of disease which they are proposed to cure.

The first case I have selected from those treated in the prison, for no other reason than that it is the only one which has occurred since my communication in the Examiner of the 15th ult. It is as follows:

*January* 22*d.*—Eliza Neill, a vagrant, comitted on the 18th inst., came under my notice today, four days after admission, under the following circumstances. Throughout yesterday, and the night previous, she exhibited some evidence of indisposition; such as disturbed sleep, low spirits, with irritable stomach and want of appetite; yet so little marked were her symptoms up to the period of my visit to the prison, that the keeper forgot, or did not think it necessary, to call my attention to her. As the day advanced, however, she became decidedly worse, and during the whole of last night was noisy and much distressed in mind. I find her to-day in a high state of nervous excitement, running around her cell in the wildest alarm, endeavouring to escape some terrific objects from which her affrighted eyes are never removed. Now and then she will suddenly stop, as if unwillingly arrested in her progress, and, with apparent effort, strive to wrest her clothes from the grasp of something, and, with a piercing shriek, supplicate to be saved from being torn to pieces. She will, at times, seem to be hedged in a corner, by the objects of her terror, and she will then resort to an ingenious and ludicrous method to elude them, by springing over their backs. In this most unhappy situation has she been since before daylight this morning, never for a moment suspending her violent exertions. The first object which excited her apprehensions was some wool, which she and her companions in confinement had been employed in picking. To her distorted vision, it had assumed the forms of hideous "wild cats," who, with open mouths, were threatening to banquet on her. The wool was immediately removed, with no abatement of her misery. Her pulse is small and frequent—tongue slightly furred and tremulous; the tremblings of her limbs are now very great. I directed

℞ Pulv. Ipecacuan. ʒi.

Tart. Antimon. gr. vi.

M. To be divided in three powders, one to be given every half hour.

*Wednesday*, 23*d.*—The emetic was given, and produced free emesis; the discharge I did not see, but learnt it was a viscid transparent fluid. After the operation of the emetic, as I have often observed where sleep does not instantly supervene, she became quieter and less disposed to move about; finally, she sank into a profound slumber, in which state I find her at this time, (12½ o'clock.) I have directed that her sleep be not disturbed, and enjoined as much quietude in the neighbourhood of her cell, as practicable.

*Thursday*, 24*th.*—My patient, I find, continued to sleep until 8 o'clock this morning, a period of from 24 to 30 hours, and then awoke with intellect unclouded, and mind serene. She commenced, with humour, a recital of the curious dreams by which she had been annoyed, and gave a laughable account of the causes of her alarm, supposing, however, that her hallucinations were but the ordinary dreams of a night. Her tongue is moist, and cleaving, and slightly tremulous; she complains of pain in her limbs, and giddiness, with weakness when she attempts to walk. Her pulse is soft and regular, though weak; there is still some tremor of limbs. I enjoined rest and light digestible diet for her. The wool has been restored to her cell, and there is little doubt, that in a few days she will be able to join her comrades at their work.

*Friday*, 25*th.*—Eliza declares herself well, though still somewhat weak; her sleep has been sound and undisturbed.

*Saturday*, 26*th.*—I have discharged my patient, cured.

The history of the second case has recently been placed in my hands, by Dr. I. Klapp of this city, and was drawn up by the late Dr. Brewster, and is in his own hand-writing. The Doctor was well known in this city as a man of much worth in his profession. He was educated in the belief of the superiority of the narcotic and stimulant treatment in delirium tremens, and of course, in his statement, could not be expected to say any thing more in behalf of the emetic practice, than what facts impelled him to do. The following is Dr. Brewster's case, and is given in his own language.

"Mr. ——, a man of intemperate habits, had suffered for several weeks under much gastric and hepatic disturbance. On Tuesday, February 22d, he was attacked with a severe paroxysm of vomiting. I saw him for the first time at about noon of that day. He was then discharging large quantities of bilious matter, and appeared very much prostrated by the great commotion into which his whole frame had been thrown. The nervous system was very much deranged, as was evinced by the irrepressible tremulousness that existed. The vomiting continued without cessation during the afternoon and succeeding night, notwithstanding the exhibition of the effervescing draught, and opium, and the application of a mustard plaster to the stomach. Towards evening some slight indications of mental aberration were observable. These increased during the night, and the next day displayed a well marked form of delirium tremens. Upon the partial subsidence of the vomiting, the use of opium and camphor was resorted to, in the proportion of a grain of the former to half a grain of the latter, every hour. Brandy and water was also given in small quantities. This treatment was continued for about fourteen hours, without any sensible effect on the disease. On the contrary, all the symptoms regularly increased, and on Thursday morning, 24th instant, Dr. Klapp was called in. The patient was found considerably excited, though very weak.* It was therefore deemed advisable to increase the quantity of brandy, and put him on the use of assafœtida and laudanum. On the following day he was wrought up to a high state of excitement, and became almost completely unmanageable. An emetic of tartarized antimony and ipecacuanha was now exhibited, and acted powerfully on the stomach. I was present during its operation, and in a few minutes, after a copious vomiting, the patient threw himself, quite exhausted, on the sofa, and went to sleep. In this state he continued for three hours, when he awoke, walked around the room a few times, and again lay down and fell into a sound sleep. This state of repose lasted the whole night, and in the morning his reason was almost entirely restored. From this moment he rapidly recovered, and in the course of two or three days was able to resume his business."

Believe me, gentlemen, to remain your much obliged and faithful friend,

W. H. KLAPP.

*Philadelphia*, February 7th, 1839.

* Generally speaking, there has been too much said about the charge of weakness from giving emetics, in this disease. No doubt cases have occurred, and will again occur, in which the depression of the system may be too great for their use, and then, if errors are committed, they must be chargeable to the faulty judgment of the physician, and not to the practice. Infallibility, or the indiscriminate use of emetics, in this disease, has *not* been contended for. The fact is, however, and it can be avouched by experience, that these delirium cases do bear the action of emetics better than any other evacuant means.

